# Associations between variants on ADIPOQ and ADIPOR1 with colorectal cancer risk: a chinese case-control study and updated meta-analysis

**DOI:** 10.1186/s12881-014-0137-y

**Published:** 2014-12-17

**Authors:** Yiyi Ou, Peizhan Chen, Ziyuan Zhou, Chenglin Li, Jinyi Liu, Kazuo Tajima, Junsheng Guo, Jia Cao, Hui Wang

**Affiliations:** Key Laboratory of Food Safety Research, Institute for Nutritional Sciences, Shanghai Institutes for Biological Sciences, Chinese Academy of Sciences, University of Chinese Academy of Sciences, Shanghai, 200031, P. R China; Medical Department, The General Hospital of Navy, Beijing, 100037, P. R China; Toxicology Institute, Key Lab of Medical Protection for Electromagnetic Radiation, Ministry of Education of China, College of Preventive Medicine; Third Military Medical University, Chongqing, 400038, P. R. China; Department of Environment Health, College of Preventive Medicine; Third Military Medical University, Chongqing, 400038, P. R. China; Division of Epidemiology and Prevention, Aichi Cancer Center Research Institute, Nagoya, Japan; Department of Military Hygiene, Faculty of Naval Medicine, Second Military Medical University, Shanghai, 200433, P. R. China; Key Laboratory of Food Safety Risk Assessment, Ministry of Health, Beijing, 100021, P. R. China; School of Life Science and Technology, ShanghaiTech University, Shanghai, 200031, P. R. China

**Keywords:** ADIPOQ, ADIPOR1, Colorectal cancer, Meta-analysis

## Abstract

**Background:**

Epidemiological studies have suggested that variants on adiponectin (ADIPOQ) and its receptor ADIPOR1 (adiponectin receptor 1) are associated with colorectal cancer (CRC) risk; however, the results were inconclusive. The aim of the study was to evaluate the associations between the variants on ADIPOQ and ADIPOR1 and the CRC risk with a hospital-based case-control study in the Chinese population along with meta-analysis of available epidemiological studies.

**Methods:**

With a hospital-based case-control study of 341 cases and 727 controls, the associations between the common variants on ADIPOQ (rs266729, rs822395, rs2241766 and rs1501299) and ADIPOR1 (rs1342387 and rs12733285) and CRC susceptibility were evaluated. Meta-analysis of the published epidemiological studies was performed to investigate the associations between the variants and CRC risk.

**Results:**

For the population study, we found that variant rs1342387 of ADIPOR1 was associated with a reduced risk for CRC [adjusted odds ratio (OR) = 0.74, 95% confidential intervals (95% CI) = 0.57-0.97; CT/TT vs. CC]. The meta-analysis also suggested a significant association for rs1342387 and CRC risk; the pooled OR was 0.79 (95% CI = 0.66-0.95) for the CT/TT carriers compared to CC homozygotes under the random-effects model (Q = 8.06, df = 4, P = 0.089; I^2^ = 50.4%). The case-control study found no significant association for variants rs266729, rs822395, rs2241766, and rs1501299 on ADIPOQ or variant rs12733285 on ADIPOR1 and CRC susceptibility, which were consistent with results from the meta-analysis studies.

**Conclusions:**

These data suggested that variant rs1342387 on ADIPOR1 may be a novel CRC susceptibility factor.

**Electronic supplementary material:**

The online version of this article (doi:10.1186/s12881-014-0137-y) contains supplementary material, which is available to authorized users.

## Background

Along with the increasing incidence of the obesity and the mortality rate of the obesity related diseases worldwide, hyperinsulinemia and insulin resistance that are likely to confer an increased risk of colorectal cancer (CRC) have drawn more attention [1]. Epidemiological studies found that several markers for insulin resistance and hyperinsulinemia, including high blood insulin, glucose, IGF-1, and C-peptide levels, are associated with the increased CRC risk [2]. Adiponectin (ADIPOQ), an insulin sensitizer secreted principally by adipocytes, is inversely associated with body fat, obesity, insulin resistance through the stimulation of insulin secretion, increment of fatty acid combustion and energy consumption [3]. Prospective studies demonstrated that an elevated adiponectin level was associated with the reduced risk of CRC in men [4], suggested that adiponectin and its downstream signaling pathways may be involved in the development of CRC.

Two adiponectin receptors (ADIPOR1 and ADIPOR2) mediate the biological activities of adiponectin in the activation of AMP-activated protein kinase activities, peroxisome proliferator-activated receptor-α and p38 mitogen-activated protein kinase, which lead to the enhanced fatty-acid oxidation in skeletal muscle cells [5]. ADIPOR1 is abundantly expressed in skeletal muscle cells, while ADIPOR2 is predominantly expressed in the liver cells [6]. Interestingly, adiponectin receptors are also found to be expressed in human malignant cells, including colorectal cancer, breast cancer, and prostate cancer *etc*., and they mediate the anticancer activities of adiponectin in the cells. *ADIPOQ* gene deficient mice showed an increased incidence of colon polyps in relative to wild-type mice when they were fed with a high-fat diet [7]. *ADIPOQ* deficiency mice also show the enhanced colorectal carcinogenesis and hepatocellular carcinoma formation activities induced by azoxymethane [8]. It was suggested that ADIPOR1 is more important than the ADIPOR2 in the regulation of the anticancer activities of ADIPOQ, as an increment in epithelial cell proliferation in ADIPOR1-deficient mice but not in ADIPOR2-deficient mice was found [7].

Since there are potential protective effects of ADIPOQ and ADIPOR1 against colorectal carcinogenesis, single nucleotide polymorphisms (SNPs) on genes *ADIPOQ* and *ADIPOR1* may contribute to the susceptibility to CRC. Kaklamani *et al*. firstly evaluated the associations between the variants of *ADIPOQ* and *ADIPOR1* with CRC risk in a two-stage case-control study [9]. They found a variant, rs266729 (C > G) on *ADIPOQ*, was associated with a reduced risk of CRC [9]. However, subsequent studies performed in other populations did not find such association [10-13]. Kaklamani *et al*. also found a significant association with CRC risk for rs822396 (*ADIPOQ*), rs822395 (*ADIPOQ*), and rs1342387 (*ADIPOR1*) in their first-stage study; however, no significant association was found for these variants in the second-stage study [9]. He *et al*. found two variants (rs1342387 and rs12733285) on *ADIPOR1* were associated with CRC risk, but not for rs266729 [12]. Liu *et al*. found no significant association for the above mentioned variants, but they identified a novel SNP, rs1063538, on *ADIPOQ* that may contribute to CRC susceptibility [13]. Other widely evaluated loci on *ADIPOQ* and their associations with the CRC risk including the rs2241766, which leads to a synonymous mutation of the amino acid for ADIPOQ protein, and rs1501299 (+276 G > T); however, no conclusive results found [9,12,13].

In the current study, we further evaluated the associations between the variants of *ADIPOQ* and *ADIPOR1* and the colorectal cancer in a southeast Chinese population. As inconsistent results were found for studies evaluated the associations between variants on *ADIPOQ* or *ADIPOR1* and CRC risk, we also performed the meta-analysis studies of the published epidemiological studies to systematically evaluate the associations between variants of *ADIPOQ* and *ADIPOR1* and the CRC risk.

## Methods

### Study populations

All the participants recruited in the current study have been described previously [14]. In briefly, a total of 341 CRC patients and 727 controls with the qualified DNA sample were included. The cases were patients who received the clinic treatments between 2001 and 2003 (aged between 30 and 80 years old) at three hospitals (Xi'nan Hospital, Xinqiao Hospital and Daping Hospital) in Chongqing City, China. All the cases were from Chongqing or the surrounding regions (including the Sichuan, Yunnan, and Guizhou provinces in the southwest of China) and histopathologically diagnosed with primary CRC for the first time within the past six months. No pre-treatment were performed at the time of recruitment for the participants. The controls were recruited from the Departments of General Surgery, Orthopedics, or Trauma who received the clinic treatments for trauma, bone fracture, appendicitis, arthritis, or varicose vein in the same hospitals. The controls were matched with the cases by age (±5 years), sex, and residence. The participants were recruited following the guidelines of the Japan, Korea, and China Colorectal Cancer Collaboration Group. The study protocol was approved by the ethics committees of the participating hospitals, including the “Ethics Committee of Xi'nan Hospital”, the “Ethics Committee of Xinqiao Hospital” and the “Ethics Committee of Daping Hospital”. All participants have provided a written informed consent and completed a structured questionnaire regarding their basic characteristics as previously reported [14].

### SNP selection and genotyping

Four most widely studied SNPs on *ADIPOQ* (including rs2241766, rs266729, rs822395 and rs1501299) and two on *ADIPOR1* (including rs12733285 and rs1342387) were selected to evaluate their associations with CRC risk. Genomic DNA was extracted with the Promega DNA Purification Wizard kit according to the manufacturer’s instructions and was stored at -20°C until use. Genotyping of the selected SNPs was performed using the Taqman-MGB probes for SNP allelic discrimination with a 7900HT Fast Real-Time PCR System (Applied Biosystems Incorporated, USA). All of the primers and probes were designed with Primer Express v3.0 (Applied Biosystems Incorporated, USA) and synthesized by the Shanghai GeneCore BioTechnologies Co., Ltd (Additional file 1 Table S1) [15]. The results were ascertained using SDS software version 2.3 (Applied Biosystems Incorporated, USA). 10% of samples were randomly selected to assess the reproducibility of the genotyping results, resulting in a more than 99% concordance.

### Meta-analysis of the associations between the selected variants with CRC risk

**Figure 1:**
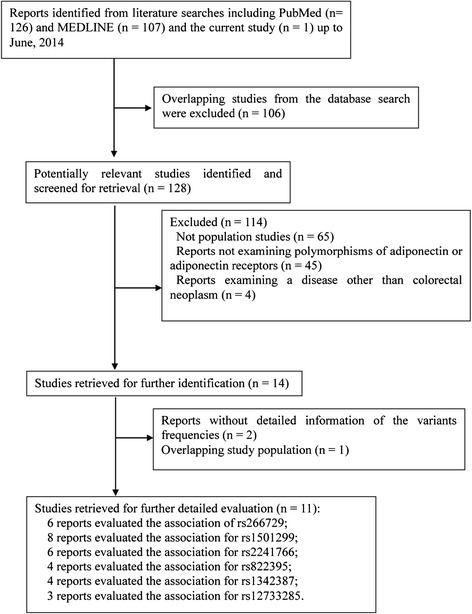
**Working flow chart for the systematic selection of the studies for meta-analysis.**

To assess the associations between the selected SNPs of ADIPOQ and ADIPOR1 with CRC risk, we performed a comprehensive and systematic search of PubMed and MEDLINE databases (updated to June, 2014), with the terms of “adiponectin,” “*ADIPOQ*,” “adiponectin receptor 1,” and “*ADIPOR1*” in combination with “colorectal cancer,” “colon cancer,” or “rectal cancer.” The goal was to identify studies that have evaluated the associations between the selected variants on the two genes and CRC risk. All the references from the identified studies were checked to identify any missing studies. The identified reports were thoroughly examined to exclude potential studies with overlapping populations. For those studies with overlapping samples, the one with the largest sample size and/or provided the detailed information about the genotype information for the participants was included in the meta-analysis. Studies were included only if they had evaluated the associations between the selected variants and CRC risk and provided sufficient information about the frequency of the genotypes in cases and controls. When there were sub-group studies or multiple study stages in the reports, they were considered as individual studies. The eligibility studies included were case-control, cohort, or cross-sectional studies that reported in the English language. The working flow chart for identification of eligible studies is shown in Figure 1.

For each report, the following information was extracted: first author, publication year, study type, study location, total number of cases and controls, and the allele frequency of the participants.

### Statistical methods

Differences in demographic characteristics were evaluated by the χ2 test (for categorical variables) and Student’s t-test (for continuous variables). The Hardy-Weinberg equilibrium was assessed by the χ2 test [one degree of freedom (d.f)] [16]. The prevalence of the genotypes for each variant was measured in cases and controls, and the associations between *ADIPOQ* or *ADIPOR1* genotypes and risk of CRC were estimated by computing odds ratios (ORs) and their corresponding 95% confidence intervals (95% CIs). The common homozygotes were recognized as references under the unconditional logistic regression statistical model with or without adjustments for covariants age, sex, drinking habit, and smoking status. The additive and the dominant genetic models were used to evaluate the associations between the variants and CRC risk. The false discovery rate (FDR) test was also performed to adjust the P-values for the multiple testing of the selected variants.

For the meta-analysis, the pooled OR and its 95% CI for the dominant genetic effect model of the selected variants were calculated using the standard inverse variance weighting method for the fixed-effects model and the DerSimonian-Laird method for the random-effects model. Heterogeneity between studies was assessed using the Cochrane’s Q-test together with the I^2^ statistic. Heterogeneity between the studies was considered significant when P < 0.1 for the Q-test or the I^2^ value was more than 25%. If significant heterogeneity between studies was found, the overall pooled estimate under the random-effects model rather than the fixed-effects model was acceptable. Publication bias was graphically represented by funnel plotting and was further assessed by Egger’s linear regression test [17]. Sensitivity analyses were conducted to evaluate the influence of individual studies on overall estimates by sequential removal of each individual study. Statistical analyses were accomplished using R Software and the SNPassoc and Meta packages (http://www.r-project.org/).

## Results

### Patient characteristics

The characteristics of the participants recruited are shown in Table 1. The mean age of the CRC cases was slightly greater than that for the control participants (P = 0.01, Table 1). Consistent with previous reports [14,18], more CRC patients had a higher daily average alcohol intake (>15 g/day) than the controls (P = 0.023, Table 1). Other potential confounders, including sex and smoking status, were not significantly different between the cases and controls (Table 1). No significant difference was also found for the percentage of the cases and controls recruited from the three hospitals (P = 0.545, Table 1).

**Table 1:** **Characteristics of the participants in the hospital based case**-**control study**

**Characteristics**	**Case (N = 341)**	**Control (N = 727)**	**P value**
Age (years,±SD)	53.7 ± 13.0	51.6 ± 11.2	0.010
Sex			
Male	184 (54.0%)	409 (56.3%)	0.481
Female	157 (46.0%)	318 (53.7%)	
Smoke			
No	216 (63.3%)	445 (61.2%)	0.503
Yes	125 (36.7%)	282 (38.8%)	
Alcohol (>15 g/d)			
No	252 (73.9%)	582 (80.1%)	0.023
Yes	89 (26.1%)	145 (19.9%)	
Recruited Center			
Xi’nan Hospital	165 (48.4%)	360 (49.5%)	0.545
Xinqiao Hospital	128 (40.5%)	282 (38.8%)	
Daping Hospital	48 (11.1%)	85 (11.7%)	

### Results for genotyping

The genotyping results of the selected SNPs in the CRC cases and controls are shown in Table 2. The overall call rate for each SNP was > 96%, and none of the selected SNPs was deviate from the Hardy-Weinberg equilibrium test (P > 0.05, Table 2). Of the six genotyped SNPs, only rs1342387 showed a significant association with CRC risk (adjusted OR = 0.80, 95% CI = 0.60-1.06 for CT vs. CC and adjusted OR = 0.59, 95% CI = 0.38-0.89 for TT vs. CC; P-trend = 0.009). Under the dominant model, the allele T carriers showed a significant decreased risk of CRC (adjusted OR = 0.74, 95% CI = 0.57-0.97; P = 0.028) in relative to the common CC carriers (Table 2). After the multiple testing corrections with the FDR method, the P value was 0.168 under the dominant genetic model and the P-trend value was 0.054 for rs1342387. For variant rs266729 of *ADIPOQ*, which may influence the circulating adiponectin levels, there was no significant association with CRC risk under any genetic model (Table 2). None of the other SNPs (rs822395, rs2241766, and rs1501299 on *ADIPOQ* or rs12733285 on *ADIPOR1*) was significantly associated with CRC risk (Table 2).

**Table 2:** **Summary data for correlation**, **call rate**, **and Hardy**-**Weinberg equilibrium test for each SNP on** ***ADIPOQ*** **and** ***ADIPOR1***

**SNP**	**Genotype**	**Cases (N %)**	**Controls (N %)**	**Crude OR (95% CI)**	**Adjusted OR** ^**a**^ **(95% CI)**	**P value** ^**a**^	**P-trend** ^**a**^	**Call rate**	**HWE-test**
rs2241766	TT	153 (47.4)	374 (52.6)	1	1		0.137	96.91%	0.462
	TG	141 (43.7)	278 (39.0)	1.24 (0.94-1.63)	1.26 (0.95-1.66)	0.109			
	GG	29 (9.0)	59 (8.3)	1.20 (0.74-1.95)	1.24 (0.76-2.02)	0.389			
	TG + GG			1.23 (0.95-1.60)	1.25 (0.96-1.64)	0.096			
rs822395	AA	226 (69.5)	501 (71.7)	1	1		0.670	96.00%	0.529
	AC	89 (27.4)	179 (25.6)	1.10 (0.82-1.49)	1.09 (0.81-1.47)	0.587			
	CC	10 (3.1)	19 (2.7)	1.17 (0.53-2.55)	1.07 (0.48-2.35)	0.870			
	AC + CC			1.11 (0.83-1.48)	1.09 (0.81-1.45)	0.582			
rs266729	CC	164 (49.0)	378 (52.1)	1	1		0.219	99.63%	0.054
	CG	152 (45.4)	305 (42.1)	1.15 (0.88-1.51)	1.18 (0.90-1.54)	0.233			
	GG	22 (5.7)	42 (5.8)	1.21 (0.70-2.09)	1.23 (0.71-2.13)	0.469			
	CG + GG			1.16 (0.89-1.50)	1.18 (0.91-1.54)	0.203			
rs1501299	GG	197 (60.6)	420 (59.2)	1	1		0.512	96.81%	0.306
	TG	110 (33.8)	244 (34.5)	0.96 (0.72-1.27)	0.95 (0.71-1.26)	0.708			
	TT	18 (5.5)	44 (6.2)	0.87 (0.49-1.55)	0.83 (0.46-1.48)	0.530			
	TG + TT			0.95 (0.72-1.24)	0.92 (0.70-1.21)	0.594			
rs12733285	CC	289 (85.5)	614 (86.0)	1	1		0.848	98.59%	0.106
	CT	47 (13.9)	93 (13.0)	1.07 (0.74-1.57)	1.03 (0.71-1.52)	0.846			
	TT	2 (0.6)	7 (1.0)	0.61 (0.13-2.94)	0.55 (0.11-2.69)	0.460			
	CT + TT			1.04 (0.70-1.53)	1.00 (0.69-1.45)	0.994			
rs1342387	CC	159 (48.0)	289 (40.5)	1	1				
	CT	135 (40.8)	312 (43.8)	0.79 (0.59-1.04)	0.80 (0.60-1.06)	0.123			
	TT	37 (11.2)	112 (15.7)	**0.60** **(0.39-** **0.91)**	**0.59** **(0.38** **-0.89)**	**0.013**	**0.009**	97.84%	0.072
	CT + TT			**0.74** **(0.57-** **0.96)**	**0.74** **(0.57-** **0.97)**	**0.028**			

^a^Adjusted for age, sex, alcohol use, and smoking status.

### Eligible studies for the meta-analysis

The working flow chart presented the selection process and the reasons for study exclusion from the meta-analysis studies (Figure 1). A total of 234 studies were initially retrieved by the database search, and 220 were excluded based on information in the titles and abstracts of the reports. For the 14 remaining, two studies that did not provide the detailed frequency data [19,20] and another study reported by Yi *et al*. [21], with the same study population reported by Kaklamani *et al*. [9], were excluded. Thus, eleven case-control studies, along with our current study, were included in our meta-analysis (Additional file 1 Tables S2-S7) [9-13,22-26].

### Results of the meta-analysis studies

**Figure 2:**
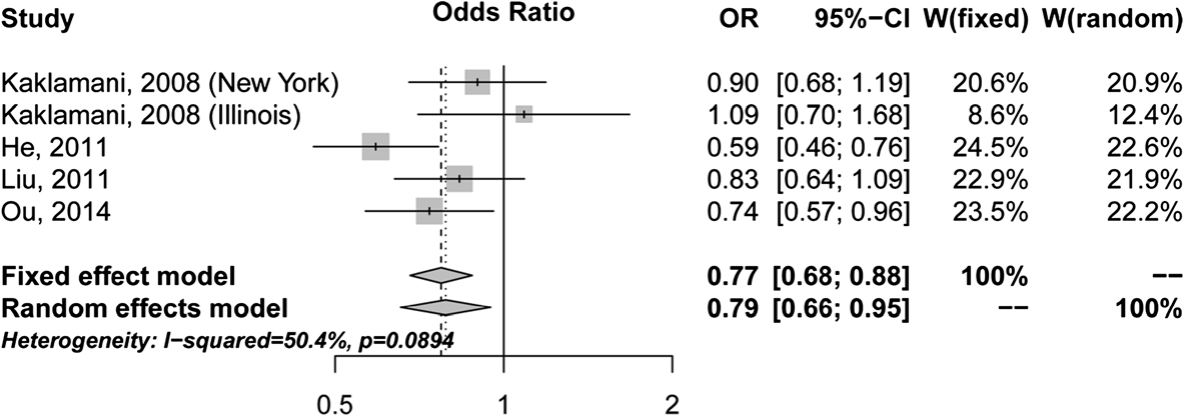
**Forest plot for the meta-analysis study between rs1342387 and CRC risk under the dominant model (CT/TT vs. CC).**

The four included reports, with five individual studies that recruited a total of 1,871 cases and 2,597 controls, have evaluated the association between variant rs1342387 and CRC risk (Additional file 1 Table S2) [9,12,13]. As determined by the meta-analysis, rs1342387 was found to be significantly associated with a reduced CRC risk, the pooled OR was 0.79 (95% CI = 0.66-0.95, P = 0.011; CT and TT vs. CC) under the random-effects model (Figure 2). Significant heterogeneity between the studies was found, as suggested by the I^2^ statistic and the Q-test (I^2^ = 50.4%; Q = 8.06, df = 4, P = 0.089; Table 3). The sensitivity analysis was performed by omitting one study at a time and recalculating the pooled ORs for the remained studies repeatedly. After excluding the study performed by He *et al*. [12], which contributed mostly to the heterogeneity between the studies, a consistent significant reduced risk for CRC was found with the pooled OR was 0.84 (95% CI = 0.73-0.98; I^2^ = 0%; Q = 2.51, df = 3, P = 0.47) for the CT and TT carriers in relative to CC carriers. No significant publication bias was found (Egger’s test, P = 0.209). Thus, the results suggested that rs1342387 may contribute to the susceptibility of CRC, which were consistent with the results of our population study.

**Table 3:** **Summary of the meta**-**analysis results for the associations between the selected variants and CRC risk**

**SNP**	**Subjects (case/control)**	**Genotype**	**Fixed**-**effects model (OR, 95% CI), p-value**	**Random**-**effects model (OR, 95% CI), p-value**	**Q-value/df**	**P value for Q test**	**I** ^**2**^	**P value of Egger’s test**
rs1342387	1871/2597	CC	1	1				
		CT	0.80 (0.70-0.92), ***0.002***	0.82 (0.67-1.00), ***0.052***	8.55/4	0.0733	53.2%	0.307
		TT	**0.71** **(0.59-** **0.86)**, < ***0.001***	**0.71** **(0.59-** **0.86)**, < ***0.001***	3.28/4	0.5114	0%	0.351
		CT + TT	**0.77** **(0.68-** **0.89)**, < **0.001**	**0.79** **(0.66-** **0.95)**, **0.011**	8.06/4	0.089	50.4%	0.209
rs266729	3635/4411	CC	1	1				
		CG	0.97 (0.79-1.19), ***0.756***	0.97 (0.77-1.22), ***0.784***	6.42/5	0.267	22.2%	0.253
		GG	0.94 (0.84-1.05), ***0.262***	0.93 (0.78-1.10), ***0.381***	11.95/5	0.036	58.2%	0.288
		CG + GG	0.95 (0.86-1.03), ***0.221***	0.93 (0.81-1.08), ***0.354***	14.45/6	0.025	58.5%	0.401
rs2241766	1519/2310	TT	1	1				
		TG	1.23 (1.08-1.40), ***0.002***	1.16 (0.92-1.46), ***0.202***	15.83/6	0.147	62.1%	0.120
		GG	1.19 (0.92-1.53), ***0.174***	1.19 (0.91-1.56), ***0.209***	6.38/6	0.382	6.0%	0.670
		TG + GG	1.22 (1.07-1.37), ***0.002***	1.18 (0.96-1.43), ***0.108***	12.93/6	0.044	53.6%	0.173
rs1501299	2749/4005	GG	1	1				
		TG	0.92 (0.82-1.02), ***0.088***	0.95 (0.82-1.05), ***0.133***	11.34/9	0.253	20.6%	0.083
		TT	0.97 (0.81-1.18), ***0.946***	0.97 (0.81-1.18), ***0.782***	7.11/8	0.524	0%	0.530
		TG + TT	0.92 (0.84-1.02), ***0.131***	0.93 (0.83-1.04), ***0.205***	10.49/9	0.312	14.2%	0.086
rs822395	1901/2639	AA	1	1				
		AC	1.01 (0.88-1.16), ***0.898***	1.00 (0.86-1.18), ***0.963***	5.38/4	0.250	25.7%	0.498
		CC	0.92 (0.72-1.17), ***0.497***	0.92 (0.72-1.17), ***0.502***	3.04/4	0.551	0%	0.032
		AC + CC	0.99 (0.87-1.13), ***0.927***	0.99 (0.87-1.13), ***0.930***	3.83/4	0.429	0%	0.793
rs12733285	1401/2139	CC	1	1				
		CT	0.86 (0.72-1.03), ***0.092***	0.86 (0.65-1.13), ***0.277***	6.81/3	0.0782	56.0%	0.841
		TT	0.96 (0.69-1.33), ***0.786***	0.96 (0.69-1.33), ***0.795***	1.45/2	0.485	0%	0.958
		CT + TT	0.87 (0.74-1.04), ***0.108***	0.87 (0.66-1.14), ***0.304***	7.12/3	0.068	57.8%	0.813

**Figure 3:**
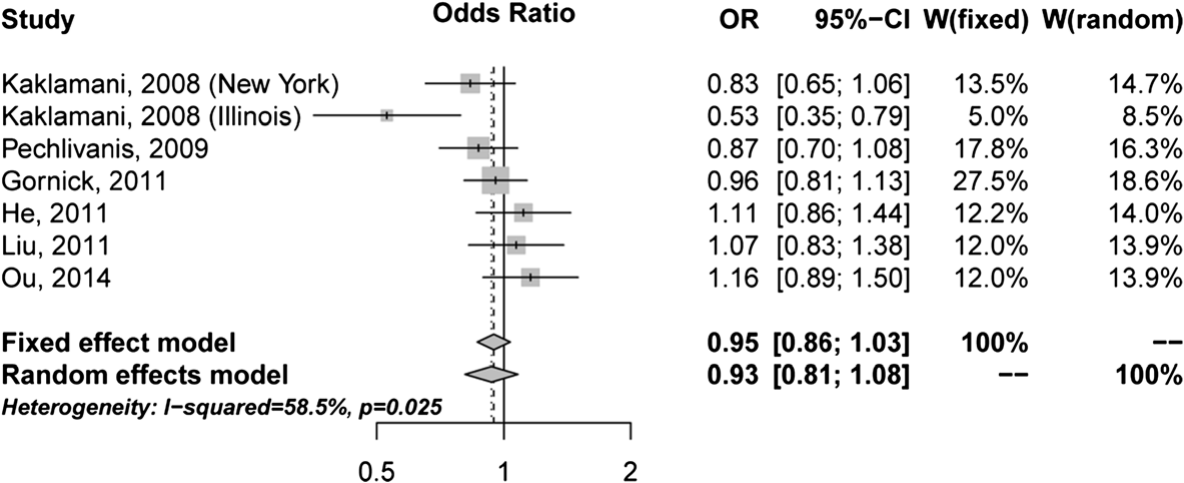
**Forest plot for the association between rs266729 with CRC under the dominant model (CG/GG vs. CC).**

rs266729 was firstly identified as a potential susceptibility locus on *ADIPOQ* for CRC (9). From the literature search, we identified six studies with seven subgroup studies that recruited a total of 3,635 cases and 4,411 controls (Additional file 1 Table S3) have evaluated the association between the rs266729 and colorectal cancer risk [9-13]. The meta-analysis showed that rs266729 did not associated with CRC risk (pooled OR = 0.93, 95% CI = 0.81-1.08, P = 0.354; CG/GG vs. CC) under the random effects model, and significant heterogeneity between the studies was found (I^2^ = 58.5%; Q = 14.45, df = 6, P = 0.025; Figure 3 and Table 3). After excluding the pioneer study conducted by Kaklamani *et al*. [9], which contributed mostly of the heterogeneity between the studies, the pooled OR was 1.00 (95% CI = 0.91-1.11; I^2^ = 0.7%; Q = 4.03, df = 4, P = 0.402) under the random-effects model. No significant publication bias was detected for the meta-analysis studies. The data indicated that rs266729 may be not a susceptibility factor for colorectal cancer.

The association of CRC risk and the other four loci were evaluated in six studies for rs2241766 (Additional file 1 Table S4), eight studies for rs1501299 (Additional file 1 Table S5), four studies for rs822395 (Additional file 1 Table S6), and three studies for rs12733285 (Additional file 1 Table S7). In our meta-analysis studies, we found no significant association between the loci and CRC risk (Table 3). The sensitivity analyses were performed to identify any individual study that significantly affects the overall estimates in the meta-analysis studies. Through sequential removal of individual study that has determined the association between the variant and CRC risk for rs822395 or rs12733285, the overall estimates for either locus did not significantly changed. However, when the study performed by Al-Harithy *et al*. [24] was excluded, which contributed mostly to the heterogeneity between the studies for rs2241766, the pooled OR was 1.26 (95% CI = 1.11-1.42; Q = 4.35, df = 5, P = 0.500, I^2^ = 0%) for the TG/GG carriers compared to TT carriers. For rs1501299, sensitivity analysis indicated that the study conducted by Tisilidis*et al*. [22] contributed mostly to the heterogeneity between the studies. The pooled OR was 0.89 (95% CI = 0.80-0.99; Q = 5.55, df = 8, p = 0.698; I^2^ = 0%) for TG/GG carriers compared to TT carriers after the study was excluded in the meta-analysis study. No significant publication bias was found, as determined by Egger’s test for all the meta-analysis studies (Table 3).

## Discussion

From the hospital-based case-control study, we found that rs1342387 on ADIPOR1 was significantly associated with a reduced CRC risk; however, no significant association with the CRC risk for variants rs266729, rs2241766, rs822395, rs1501299, and rs12733285 was found. As determined by the meta-analysis of published epidemiological studies, only variant rs1342387 was significantly associated with the reduced CRC susceptibility, whereas limited data supported the associations between the other loci and CRC susceptibility. As the consistent results were found for the selected variants between hospital based case-control studies and the meta-analysis studies, which suggested that rs1342387 is associated with a reduced risk for colorectal cancer. Our results appeared to provide a genetic relationship between obesity, hyperinsulinemia, and/or insulin resistance and the risk of CRC.

Variant rs1342387 was associated with a reduced CRC risk in the Chinese population, which is consistent with the study conducted by He *et al*. [12] and the first-stage study of Kaklamani *et al*. [9]. Although the P value for multiple testing with the FDR method suggested a statistically non-significant association between the rs1342387 and CRC risk, which could be due to the high linkage disequilibrium (LD) between the variants, the results from the meta-analysis studies provided stronger evidence that the allele T carriers were associated with a significant reduced risk of CRC in relative to CC homozygotes. rs1342387 is located in intron 4 (+5843 C > T) of the *ADIPOR1* gene, a locus associated with body size measurements, such as weight, height, waist and hip circumference, sagittal diameter, and body mass index [27]. Another study, performed in the Amish population, found that allele T for variant rs1342387 was associated with a reduced risk for type 2 diabetes, although the possibility that other functional loci in high linkage-disequilibrium with rs1342387 may account for this association could not be excluded [29]. Crimmins *et al*. summarized the association between the variants of *ADIPOR1* and *ADIPOR2* with insulin resistance and type 2 diabetes, and they found that only rs1342387 was significantly associated with the reduced risk for insulin resistance [30]. Since insulin resistance and type 2 diabetes may lead to susceptibility to various types of cancer, the variant may be a susceptibility factor for CRC and other obesity-related cancers.

The variant rs266729 has been reported to be associated with higher circulating adiponectin levels [31,32], higher plasma total antioxidant status (32), lower plasma oxidized-LDL levels [33], and reduced risk for type 2 diabetes [34]. Other studies, however, did not find an association for the variant with blood lipids [35], blood pressure [35], or with coronary heart disease risk [36]. There are no significant allelic specific effects of promoter activity for rs266729 alone [37], and rs266729 may act together with other loci to regulate the activity of the adiponectin promoter [38]. In their first-stage study, Kaklamani *et al*. reported a significant association for the locus and CRC risk in a population of Ashkenazi origin. In their second-stage study, a significant association between the variant and CRC risk in a population of mixed origins was also noticed [9]. However, subsequent studies found no such association, except for one study conducted by He *et al*. have reported that the variant was associated with colon cancer susceptibility but not rectal cancer [12]. Meta-analysis of the epidemiological studies suggested that the locus does not contribute to CRC susceptibility. The original study may represent a chance observation. However, the possibility that the association observed by Kaklamani *et al*. [9] is attributed to the population-specific effects for rs266729 on CRC risk cannot be excluded, as the subsequent studies were conducted in other countries or races. There is also a possibility that the variant acts together with other variants to influence the CRC risk, and the genetic background may influence the association of the variant and CRC risk. Thus, more studies with fine-mapping methods, are warranted to address these questions.

For the other four variants, we found no significant association with CRC risk in our hospital based case-control population study, which was consistent with the results of the meta-analysis studies. For variant rs2241766, which is located on exon 2 of *ADIPOQ*, the G to T allelic change leads to a synonymous variation of the amino acid (Gly to Gly). The meta-analysis study found no significant association between the variant and CRC risk; however, the sensitivity study suggested that the results may be affected by the heterogeneity between the studies. rs1501299 is located on intron 2 (+276 G > T) of *ADIPOQ* and the meta-analysis conducted by Xu *et al*. found a decreased CRC risk for GT and TT carriers compared to GG carriers; however, only four studies were included [39]. For the current meta-analysis study, based on eight studies, suggested that the locus is not associated with CRC risk; however, when the study performed by Tisilidis *et al*. [22], which contributed mostly to the heterogeneity between the studies was excluded, the pooled estimate suggested that the variant may be a protective factor for CRC. Whether rs2241766 and rs1501299 are associated with the CRC susceptibility should be determined with more studies of relatively larger sample size. Variant rs822395 is located on intron 1 of *ADIPOQ*, and rs12733285 is located on intron 1 of *ADIPOR1*. Neither variant showed a significant association with CRC risk in our population study or in the meta-analysis studies.

We acknowledged that there were several limitations for the current study. Firstly, the investigation was a hospital based case-control study, which is more prone to selection bias of the participants. The age of the control subjects was slightly younger than that for the CRC cases, and the CRC risk for the controls was difficult to determine. Secondly, the relatively small sample size may lead to a lower statistical power to detect the association between rs266729 and other variants with CRC risk. Lastly, the underlying mechanisms for the association between rs1342387 and CRC risk need to be elucidated.

## Conclusions

In conclusion, variant rs1342387 of *ADIPOR1* may contribute to CRC susceptibility. Results from our population study and meta-analysis suggest that variants rs266729, rs822395, rs2241766, and rs1501299 of *ADIPOQ* and variant rs12733285 of *ADIPOR1* may not contribute to CRC susceptibility. However, more investigations with larger sample sizes are warranted to validate these results and the underlying mechanisms are also need to be elucidated.

## Additional file

Additional file 1: Table S1. Information on primers and probes used for genotyping of the six variants. **Table S2.** Studies included in the meta-analysis for the association between rs1342387 and CRC. **Table S3.** Studies included in the meta-analysis for the association between rs266729 and CRC. **Table S4.** Studies included in the meta-analysis for the association between rs2241766 and CRC. **Table S5.** Studies included in the meta-analysis for the association between rs1501299 and CRC. **Table S6.** Studies included in the meta-analysis for the association between rs822395 and CRC. **Table S7.** Studies included in the meta-analysis for the association between rs12733285 and CRC.
