# Supplementary material for: Associations between variants on ADIPOQ and ADIPOR1 with colorectal cancer risk: a chinese case-control study and updated meta-analysis
Source: BMC Med Genet. 2014 Dec 17;15:137. doi: 10.1186/s12881-014-0137-y (PMC4411774; doi:10.1186/s12881-014-0137-y)
Supplement: Additional file 1: Table S1. — Information on primers and probes used for genotyping of the six variants. Table S2. Studies included in the meta-analysis for the association between rs1342387 and CRC. Table S3. Studies included in the meta-analysis for the association between rs266729 and CRC. Table S4. Studies included in the meta-analysis for the association between rs2241766 and CRC. Table S5. Studies included in the meta-analysis for the association between rs1501299 and CRC. Table S6. Studies included in the meta-analysis for the association between rs822395 and CRC. Table S7. Studies included in the meta-analysis for the association between rs12733285 and CRC. [file 12881_2014_137_MOESM1_ESM.docx]

**Supplementary Tables**

**Supplementary Table 1.** Information on primers and probes used for genotyping of the six variants.

**Supplementary Table 2.** Studies included in the meta-analysis for the association between rs1342387 and CRC.

**Supplementary Table 3.** Studies included in the meta-analysis for the association between rs266729 and CRC.

**Supplementary Table 4.** Studies included in the meta-analysis for the association between rs2241766 and CRC.

**Supplementary Table 5.** Studies included in the meta-analysis for the association between rs1501299 and CRC.

**Supplementary Table 6.** Studies included in the meta-analysis for the association between rs822395 and CRC.

**Supplementary Table 7.** Studies included in the meta-analysis for the association between rs12733285 and CRC.

**Supplementary Table 1.** Information on primers and probes used for genotyping of the six variants.

| **SNP** | **Primer sequence** | **Taqman probe sequence** | |
| --- | --- | --- | --- |
| rs266729  (C > G) | F: TTCATCAGAATGTGTGGCTTGC | P1 | FAM-ATCCTGCCCTTCAA-TAMRA |
|  | R: GCAACATTCAACACCTTGGACTT | P2 | HEX-ATCCTGCCCTTCAA-TAMRA |
| rs822395  (A > C) | F: CCTACCCTAAGGGAACACTGTTTGT | P1 | FAM-TATGGACATATTTCTCC-TAMRA |
|  | R: CCACTCTTGTATTTTTGGCACCTC | P2 | HEX-TATGGACAGATTTCTC-TAMRA |
| rs1501299  (G > T) | F: TTTCATCACAGACCTCCTACACTGA | P1 | FAM-TATATGAAGGCATTCATTA-TAMRA |
|  | R: AGATGCAGCAAAGCCAAAGTC | P2 | HEX-TATATGAAGTCATTCATTATT-TAMRA |
| rs2241766  (T > G) | F: CCATGGCTGACAGTGCACAT | P1 | FAM-TCTGCCCGGTCATGACCAGGA-TAMRA |
|  | R: GCCCGCCATCCAACCT | P2 | HEX-TCTGCCCGGGCATGACCAG-TAMRA |
| rs12733285  (C > T) | F: ACACATCAGGTAAGGGCAATCC | P1 | FAM-CCACAAGCACAGTTG-TAMRA |
|  | R: TTGACACCATATAAAGGTCTGCTGTAT | P2 | HEX-CCACAAGCATAGTTG-TAMRA |
| rs1342387  (C>T) | F: CCCTGATGATTAGTGATGTTGCC | P1 | FAM-ACATCAACCATCAAAGT-TAMRA |
|  | R: GAGAAACAGCACGAAACCTGC | P2 | HEX-CATCAACCGTCAAAGT-TAMRA |

**Supplementary Table 2.** Studies included in the meta-analysis for the association between rs1342387 and CRC.

| Study  ( First author, year ) | Location | Study type | Sample size (case/control) | Genotype distribution (case/control) | | | Ref |
| --- | --- | --- | --- | --- | --- | --- | --- |
|  |  |  |  | CC | CT | TT |  |
| Kaklamani, 2008 | New York, USA | Population based Case-control | 441/658 | 113/155 | 223/313 | 99/179 | [9] |
|  | Illinois, USA | Hospital based Case-control | 199/199 | 57/61 | 101/99 | 32/32 |  |
| He, 2011 | China | Population based Case-control | 420/555 | 213/210 | 157/263 | 50/82 | [12] |
| Liu, 2011 | China | Hospital based Case-control | 470/458 | 189/165 | 222/227 | 56/64 | [13] |
| Ou, 2014 | China | Hospital based Case-control | 341/727 | 159/289 | 135/312 | 37/112 |  |

**Supplementary Table 3.** Studies included in the meta-analysis for the association between rs266729 with CRC.

| Study  ( First Author, Year ) | Location | Study type | Sample size (case/control) | Genotype distribution (case/control) | | | Ref |
| --- | --- | --- | --- | --- | --- | --- | --- |
|  |  |  |  | CC | CG | GG |  |
| Kaklamani, 2008 | New York, USA | Population based Case-control | 441/658 | 244/340 | 163/271 | 27/47 | [9] |
|  | Illinois, USA | Hospital based Case-control | 199/199 | 112/82 | 77/103 | 6/12 |  |
| Pechlivanis, 2009 | CzechRepublic | Hospital based Case-control | 702/752 | 366/373 | 238/278 | 55/65 | [10] |
| Gornick, 2011 | Israel | Population based Case-control | 1062/1062 | 588/576 | 474/486 | | [11] |
| He, 2011 | China | Population based Case-control | 420/555 | 173/243 | 205/261 | 42/51 | [12] |
| Liu, 2011 | China | Hospital based Case-control | 470/458 | 237/240 | 184/179 | 44/37 | [13] |
| Ou, 2014 | China | Hospital based Case-control | 341/727 | 164/378 | 152/305 | 22/42 |  |

**Supplementary Table 4.** Studies included in the meta-analysis for the association between rs2241766 and CRC.

| Study  ( First Author, Year ) | Location | Study type | Sample size (case/control) | Genotype distribution (case/control) | | | Ref |
| --- | --- | --- | --- | --- | --- | --- | --- |
|  |  |  |  | TT | TG | GG |  |
| Kaklamani, 2008 | New York, USA | Population based Case-control | 441/658 | 279/435 | 141/172 | 20/41 | [9] |
|  | Illinois, USA | Hospital based Case-control | 199/199 | 151/164 | 46/31 | 2/0 |  |
| Partida-Perez, 2010 | Mexico | Hospital based Case-control | 58/111 | 42/74 | 16/34 | 0/3 | [23] |
| He, 2011 | China | Population based Case-control | 420/555 | 190/278 | 193/238 | 37/39 | [12] |
| Al-Harithy, 2012 | Saudi Arabia | Hospital based Case-control | 60/60 | 40/27 | 14/32 | 6/1 | [24] |
| Hu, 2013 | Sichuan, China | Population based Case-control | 400/400 | 173/208 | 196/165 | 31/27 | [26] |
| Ou, 2014 | China | Hospital based Case-control | 341/727 | 153/374 | 141/278 | 29/59 |  |

**Supplementary Table 5.** Studies included in the meta-analysis for the association between rs1501299 and CRC.

| Study  ( First Author, Year ) | Location | Study type | Sample size (case/control) | Genotype distribution (case/control) | | | Ref |
| --- | --- | --- | --- | --- | --- | --- | --- |
|  |  |  |  | GG | TG | TT |  |
| Kaklamani, 2008 | New York, USA | Population based Case-control | 441/658 | 208/285 | 180/293 | 45/58 | [9] |
|  | Illinois, USA | Hospital based Case-control | 199/199 | 105/94 | 78/85 | 15/17 |  |
| Tsilidis, 2009 | Washington, USA | Population based Case-control | 208/381 | 96/198 | 86/134 | 19/27 | [22] |
| Partida-Perez, 2010 | Mexico | Hospital based Case-control | 58/94 | 30/51 | 25/40 | 3/3 | [23] |
| He, 2011 | China | Population based Case-control | 420/555 | 220/265 | 160/224 | 40/66 | [12] |
| Liu, 2011 | China | Hospital based Case-control | 470/458 | 262/226 | 167/201 | 36/29 | [13] |
| Al-Harithy, 2012 | Saudi Arabia. | Hospital based Case-control | 60/60 | 55/58 | 5/2 | 0/0 | [24] |
| Keku, 2012 | North Carolina, USA(Whites) | Population based Case-control | 312/545 | 122/223 | 106/204 | 27/36 | [25] |
|  | North Carolina, USA (African) | Population based Case-control | 240/328 | 78/116 | 94/119 | 19/41 |  |
| Ou, 2014 | China | Hospital based Case-control | 341/727 | 197/420 | 110/244 | 18/44 |  |

**Supplementary Table 6.** Studies included in the meta-analysis for the association between rs822395 and CRC.

| Study  ( First Author, Year ) | Location | Study type | Sample size (case/control) | Genotype distribution (case/control) | | | Ref |
| --- | --- | --- | --- | --- | --- | --- | --- |
|  |  |  |  | AA | AC | CC |  |
| Kaklamani, 2008 | New York, USA | Population based Case-control | 441/658 | 184/301 | 202/265 | 45/88 | [9] |
|  | Illinois, USA | Hospital based Case-control | 199/199 | 76/77 | 89/97 | 28/22 |  |
| Gornick, 2011 | Israel | Population based Case-control | 500/500 | 223/205 | 222/230 | 55/65 | [11] |
| He, 2011 | China | Population based Case-control | 420/555 | 343/440 | 70/109 | 7/6 | [12] |
| Ou, 2014 | China | Hospital based Case-control | 341/727 | 226/501 | 89/179 | 10/19 |  |

**Supplementary Table 7.** Studies included in the meta-analysis for the association between rs12733285 and CRC.

| Study  ( First Author, Year ) | Location | Study type | Sample size (case/control) | Genotype distribution (case/control) | | | Ref |
| --- | --- | --- | --- | --- | --- | --- | --- |
|  |  |  |  | CC | CT | TT |  |
| Kaklamani, 2008 | New York, USA | Population based Case-control | 441/658 | 147/200 | 221/347 | 69/105 | [9] |
|  | Illinois, USA | Hospital based Case-control | 199/199 | 98/101 | 78/77 | 19/14 |  |
| He, 2011 | China | Population based Case-control | 420/555 | 386/477 | 34/78 | 0/0 | [12] |
| Ou, 2014 | China | Hospital based Case-control | 341/727 | 289/614 | 47/93 | 2/7 |  |
